# Effects of simulated microgravity on the expression profiles of RNA during osteogenic differentiation of human bone marrow mesenchymal stem cells

**DOI:** 10.1111/cpr.12539

**Published:** 2018-11-05

**Authors:** Liang Li, Cui Zhang, Jian‐ling Chen, Fan‐fan Hong, Ping Chen, Jin‐fu Wang

**Affiliations:** ^1^ Institute of Cell and Development Biology, College of Life Sciences Zhejiang University Hangzhou China; ^2^ Departments of Cell Biology and Otolaryngology Emory University School of Medicine Atlanta Georgia

**Keywords:** expression profile of RNA, hBMSCs, osteogenic differentiation, RNA‐seq, simulated microgravity

## Abstract

**Objectives:**

Exposure to microgravity induces many adaptive and pathological changes in human bone marrow mesenchymal stem cells (hBMSCs). However, the underlying mechanisms of these changes are poorly understood. We revealed the gene expression patterns of hBMSCs under normal ground (NG) and simulated microgravity (SMG), which showed an interpretation for these changes by gene regulation and signal pathways analysis.

**Materials and methods:**

In this study, hBMSCs were osteogenically induced for 0, 2, 7 and 14 days under normal ground gravity and simulated microgravity, followed by analysis of the differences in transcriptome expression during osteogenic differentiation by RNA sequencing and some experimental verification for these results.

**Results:**

The results indicated that 837, 399 and 894 differentially expressed genes (DEGs) were identified in 2, 7 and 14 days samples, respectively, out of which 13 genes were selected for qRT‐PCR analysis to confirm the RNA‐sequencing results. After analysis, we found that proliferation was inhibited in the early stage of induction. In the middle stage, osteogenic differentiation was inhibited, whereas adipogenic differentiation benefited from SMG. Moreover, SMG resulted in the up‐regulation of genes specific for tumorigenesis in the later stage.

**Conclusion:**

Our data revealed that SMG inhibits the proliferation and inhibits the differentiation towards osteoblasts but promotes adipogenesis. SMG also selects highly tumorigenic cells for survival under prolonged SMG.

## 1 INTRODUCTION

Previous studies have shown that microgravity induces significant bone loss in weight‐bearing bones at a rate of approximately 1%‐2% per month.^1^ Bone loss under microgravity may be due to the inhibition of osteoblast development and the increase in osteoclastic activity.^2^ Exposure to microgravity for a few days has shown a marked suppression of differentiation of human bone marrow mesenchymal stem cells (hBMSCs) into osteoblasts detected by a decrease in *ALPL*,* COL1A1*,* SPARC* and *RunX2*.^3^ Under microgravity, osteoblasts show shorter and wavier microtubules, smaller and fewer focal adhesions, and thinner cortical actin and stress fibres. Simultaneously, microgravity causes an increase in bone resorption by osteoclasts and a decrease in osteoblast cellular integrity.^4^ Microgravity also has a significant effect on cell proliferation. Mouse embryonic stem (ES) cells cultured under the SMG condition had a significantly reduced total cell number compared to cells cultured under normal ground gravity (NG).^5^ Another study found that the proliferation of murine bone marrow stromal cells was also inhibited in space flight.^6^ It has been reported that cells under SMG are more flattened and reached confluence at a lower cell density.^7^

Random positioning machine (RPM) is a kind of 3D clinostat that provides continuous random changes in orientation relative to the gravity vector, which can be used to simulate microgravity.^8^ In this study, we used an RPM to simulate microgravity and evaluated the effects of SMG on whole gene expression during osteogenesis of hBMSCs. We performed an RNA‐seq analysis of hBMSCs induced osteogenically under SMG and NG, and clustered differentially expressed genes (DEGs) into several functional groups to analyse the impacts of SMG on the physiology of hBMSCs.

## 2 MATERIALS AND METHODS

### 2.1 Preparation of hBMSCs

This experiment was performed in accordance with standard ethical guidelines and approved by the Institutional Review Board and the Ethical Committee of the First Affiliate Hospital, Zhejiang University, China. Informed consent was obtained from all the healthy donors: one male donor aged 23 and two female donors aged 19 and 34. Whole bone marrow samples were collected at the First Affiliate Hospital, and hBMSCs were isolated, cultured and passaged as previously described.^9^ Cells of 3rd passage were used for the experiments, and the cells examined for the expression profile of special surface antigens expressed CD19^−^, CD34^−^, CD14^−^, CD45^−^, HLA^−^, DR^−^, CD105^+^, CD90^+^, CD73^+^ and CD29^+^ (Figure S1). In addition, these cells had osteogenic and adipogenic potentials indicating that the cells prepared from human bone marrow can be accepted as hBMSCs (Figure S2).^10^, ^11^

### 2.2 Microgravity simulation and induction with random position machine

The SM‐31 random position machine (RPM) (Chinese Academy of Science, Beijing, China) was used to simulate microgravity condition. It was operated in random modes of speed (0.1‐10 rpm) inside a CO_2_ incubator (5% CO_2_, 37°C and 100% humidity). After the cells of 3rd passage reached 60%‐70% confluency, the medium was replaced with osteogenic medium^12^ (L‐Dulbecco's modified Eagle's medium (DMEM; Life Technologies, Shanghai, China) supplemented with 10% FBS, 50 μg/mL l‐ascorbic acid (Sigma, Shanghai, China), 10 mmol/L β‐glycerophosphate (Sigma), 0.1 μmol/L dexamethasone (Sigma), 100 U/mL penicillin and 100 μg/mL streptomycin. The flasks were fixed on a cell culture vessel for RPM exposure. Cells grown in NG condition were also filled with osteogenic medium and were statically placed in the same CO_2_ incubator. The medium was changed every other day. Cells induced for 0, 2, 7 and 14 days were used to study the effects of SMG. The subsequent results follow these time‐points unless otherwise specified.

### 2.3 RNA extraction, sequencing and analysis

RNA from cells was extracted using Trizol reagent (Sigma). All RNA samples were detected and analysed in Vazyme Biotech Co. (Nanjing, China). RNA integrity and concentration were detected by 1.2% agarose gel electrophoresis and Agilent 2100 Bioanalyzer. cDNA libraries were constructed with rRNA elimination and sequenced by the Illumina Hiseq X Ten platform as 150‐bp pair‐ended reads. Reads were aligned using Hisat2 (v2.0.5, Center for Computational Biology, Johns Hopkins University, Baltimore, MD, USA). FPKM estimation was performed with Cuffdiff (v1.3.0, Cole Trapnell’s lab at the University of Washington, Seattle, WA, USA), aligned reads were counted with HTSeq and differential expression analysis was performed with Cuffdiff (v2.2.1). Differentially expressed genes were selected using a cut‐off at a P value of less than 0.05 (FDR adjusted for multiple testing) and the absolute value of log2 (fold change) greater than or equal to 1. The data discussed in this publication have been deposited in NCBI's Gene Expression Omnibus^13^, ^14^ and are accessible through GEO Series accession number GSE114117 (https://www.ncbi.nlm.nih.gov/geo/query/acc.cgi).

### 2.4 Validation of gene expression by qRT‐PCR

Reverse transcription of 1 μg of total RNA was performed using the RevertAid First Strand cDNA Synthesis Kit (Thermo Scientific, Shanghai, China). Primers (Table S1) were designed using Primer Premier 6.0 Demo (PREMIER Biosoft, Palo Alto, CA, USA) and Oligo 7.36 Demo software (Molecular Biology Insights, Inc., Colorado Springs, CO, USA). Quantitative real‐time PCR reactions using the LightCycler® 480 SYBR Green qPCR Supermix (Roche, Shanghai, China) were performed in the CFX96 Touch Deep Well Real‐Time PCR Detection System (Bio‐Rad, Berkeley, CA) with 18S rRNA as an internal control. The thermal cycler protocol included denaturation at 95°C for 1 minute followed by 40 cycles of 95°C for 10 second, 56°C for 30 second and 72°C for 20 second. Quantification of selected gene expression was performed using the relative quantitation (2^−ΔΔCT^) method. All experiments were performed in triplicate, and three independent experiments were performed.

### 2.5 Cell proliferation assay and cell cycle analysis by PI staining

Cell proliferation at 0, 12, 24 and 48 hour of osteogenic induction was evaluated using the Cell Counting Kit‐8 (Qi Hai, Hangzhou, China) according to the manufacturers’ instructions. Each sample was measured in triplicate wells. The absorbance of the reaction solution was measured at 450 nm. Cell cycle was detected by Cell Cycle and Apoptosis Analysis Kit (Beyotime, Shanghai, China) according to the manufacturers’ instructions. The percentage of cells in G0/G1, S and G2/M stage were determined by Cytomics^TM^ FC500 Flow Cytometry (Beckman Coulter Inc., Shanghai, China).

### 2.6 Immunohistochemistry assays

The analysis of ALP activity was performed using cells osteogenically induced for 2, 7 and 14 days. ALP activity was analysed using the ALP Quantitative Analysis Kit (Nanjing Jiancheng Institute, China) according to the manufacturers’ instructions. The ALP activity was determined at 405 nm using ARM‐100 Microplate Reader (Allsheng, Hangzhou, China). In addition, cells induced for 2, 7 and 14 days were also stained with Oil Red O as previously described.^15^ For immunolabelling of cytoskeleton, Filamentous actin (F‐actin) was stained with phalloidin‐TRITC (P1951; Sigma), diluted with 1:300 and nuclei with 2 µg/mL 4′,6‐diamidino‐2‐phenylindole (DAPI) in PBS for 10 minute. Images were obtained using ZEISS LSM 710 NLO Multiphoton microscope (Shanghai, China).

### 2.7 Statistical analysis

All values are expressed as the mean ± standard deviation (SD). Statistical analysis was performed using a two‐tailed unpaired Student's *t* test or analysis of variance (ANOVA) for multiple comparisons. *P* < 0.05 was considered statistically significant and *P* < 0.01 as highly significant.

## 3 RESULTS

### 3.1 Sequencing results and quality control

Paired‐end RNA‐Seq reads of 150 bp were generated by using the VAHTS mRNA‐seq v2 Library Prep Kit. Mapping the sequence reads onto the human genome revealed that a total of 356 143 952 raw reads were generated from all the cDNA libraries. After removing adapters, low‐quality regions, and possible contamination, 353 406 202 clean reads with an average GC of 51.98% were obtained. The proportion of clean reads in the human transcriptome libraries that mapped to the human reference genome ranged from 89.90% to 91.14%, while the adapter proportion ranged from 0.48% to 0.97%. The proportion of reads with a Phred quality value Q > 30 among the clean reads ranged from 90.64% to 91.01%. Mapping of exons, introns and intergenic regions to the human reference genome accounted for an average of 91.09%, 6.87% and 2.04%, respectively. The base quality distribution of all samples is shown in Table S2.

### 3.2 Global RNA‐seq data analysis

**Figure 1:**
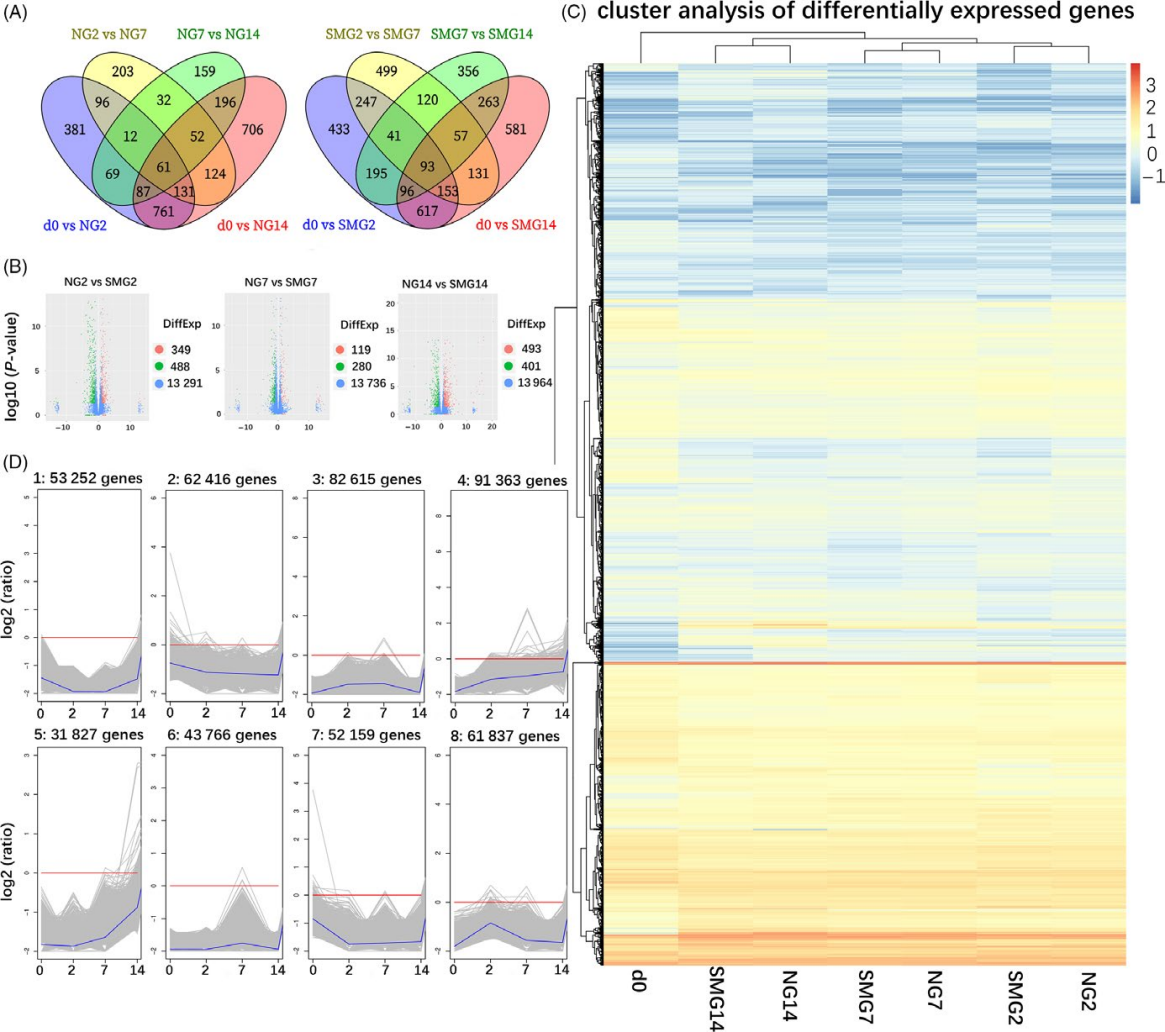
DEGs of days 2, 7 and 14 and clustering of DEGs. (A) Venn diagram of DEGs comparing d0 vs NG2, NG2 vs NG7, NG7 vs NG14, and d0 vs NG14 and Venn diagram of DEGs comparing d0 vs SMG2, SMG2 vs SMG7, SMG7 vs SMG14 and d0 vs SMG14. (B) DEGs of NG2 vs SMG2, NG7 vs SMG7 and NG14 vs SMG14. The red, green and blue dots represent up‐regulated, down‐regulated and not significant genes, respectively. (C) The heatmap shows the K‐means clustering of transformed expression values for mRNAs. Yellow represents higher expression, and blue represents lower expression. (D) The line charts represent the expression patterns of genes in the NG and SMG group in eight clusters corresponding to the heatmap. The first four clusters are in the NG group, and the last four clusters are in the SMG group. DEGs, differentially expressed genes; NG, normal ground condition; SMG, simulated microgravity; d0, cells induced for 0 d; NG2, cells induced for 2 d under normal ground condition; SMG2, cells induced for 2 d under simulated microgravity; NG7, cells induced for 7 d under normal ground condition; SMG7, cells induced for 7 d under simulated microgravity; NG14, cells induced for 14 d under normal ground condition; SMG14, cells induced for 14 d under simulated microgravity

Differentially expressed genes (DEGs) were obtained by pairwise comparisons of samples collected from cells induced for days 0 (d0), 2 (NG2, SMG2), 7 (NG7, SMG7) and 14 (NG14, SMG14), respectively. The types and numbers of DEGs varied with induction time. As shown in Figure 1A, 61 DEGs were common in NG group and 93 DEGs in SMG group when the four induction points were compared. In total, 837 DEGs were detected in NG2 vs SMG2 samples (349 DEGs up‐regulated and 488 DEGs down‐regulated), 399 DEGs in NG7 vs SMG7 samples (119 DEGs up‐regulated and 280 DEGs down‐regulated) and 894 DEGs in NG14 vs SMG14 samples (493 DEGs up‐regulated and 401 DEGs down‐regulated) (Figure 1B).

K‐means clustering of all DEGs using the Euclidean distance method was performed. Hierarchical clustering of 5411 DEGs (Figure 1C) exhibited similar expression patterns in the principal component analysis. The expression profiles of d0 were different from the other profiles; NG2 and SMG2, NG7 and SMG7, NG14 and SMG14 clustered together. Similarity between groups of day 2 and day 7 was more compared to those of day 14. In comparison with NG and SMG, we found that most genes in cells under SMG2 and SMG7 had a down‐regulated expression pattern (more blue bands) compared with those in cells under NG2 and NG7, respectively. In NG14 vs SMG14, there was no obvious change in the number of up‐ and down‐regulated genes. However, the genes up‐ or down‐regulated were different between cells under NG14 and SMG14. Eight main clusters were plotted with expression patterns of genes (Figure 1D). We selected the DEGs in each cluster and classified them based on UniProtKB keywords (Table S3). The first four clusters were in the NG group and the last four clusters were in the SMG group. Cluster 1 represents genes whose expression level decreased from day 0 to day 2 and then increased from day 7 to day 14. DEGs included in cluster 1 were mainly enriched in disulphide bond and signal. Cluster 2 represents genes that underwent an overall trend of decrease, and DEGs were mainly enriched in developmental protein and glycoprotein. Cluster 3 shows genes up‐regulated from day 0 to day 2 and down‐regulated from day 7 to day 14, in contrast to cluster 1. No DEG was included in cluster 3. Clusters 4 represents genes that underwent an overall trend of increase, and DEGs were mainly enriched in glycoprotein and signal. Cluster 5 shows the same trend as cluster 4, and DEGs were mainly enriched in transmembrane helix and membrane. Cluster 6 represents genes up‐regulated at day 7 and down‐regulated at day 14, and no DEG was included in this cluster. Cluster 7 shows genes with decreased expression at day 2 and no significant change at day 7 and day 14. DEGs were mainly enriched in signal and serine protease inhibitor. Cluster 8 represents genes up‐regulated on day 2 and down‐regulated on days 7 and 14, and DEGs were mainly enriched in metal‐binding and microsome.

### 3.3 Gene ontology and Kyoto encyclopaedia of genes and genomes enrichment analysis of differentially expressed genes

**Figure 2:**
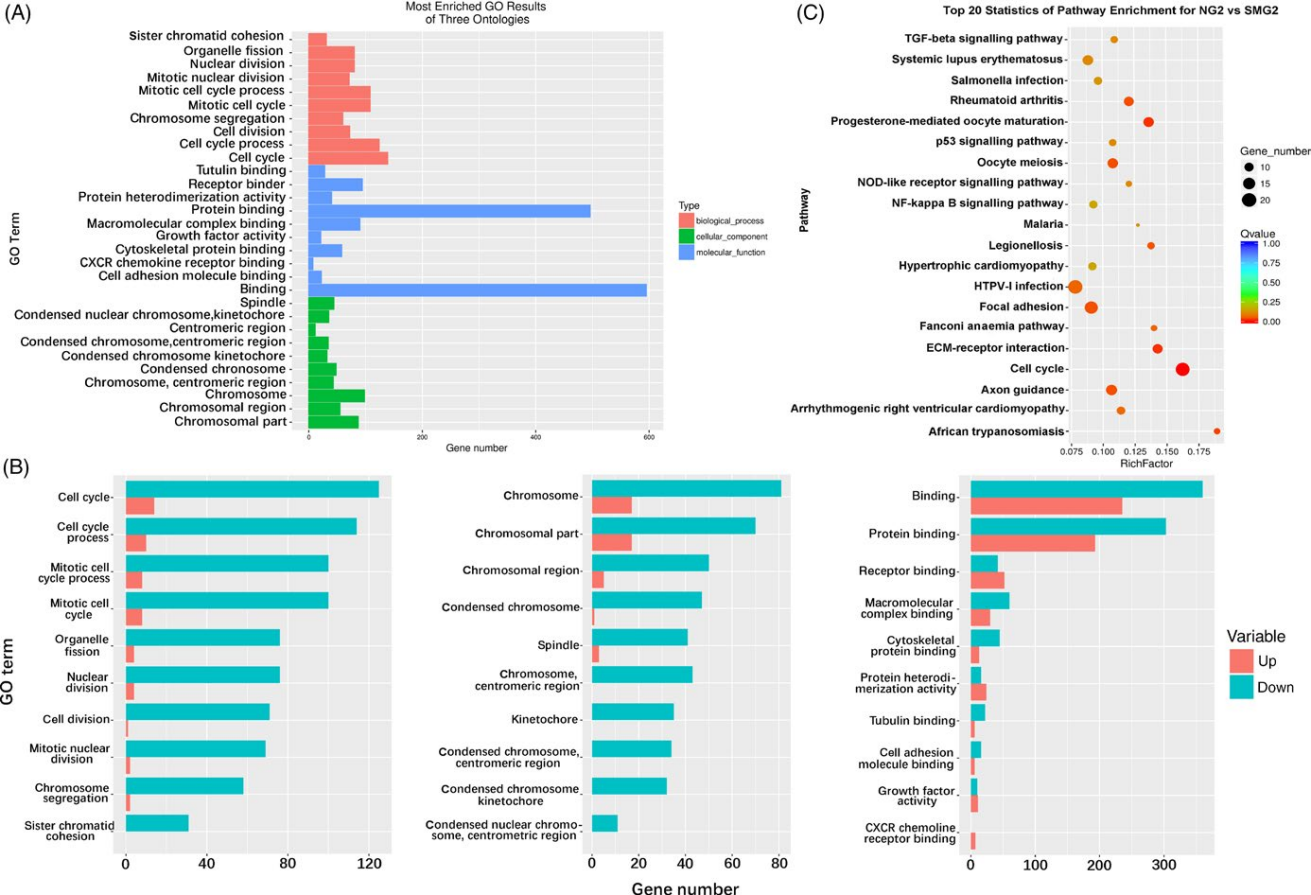
GO analysis of DEGs in three ontologies and Pathway analysis of DEGs in NG2 vs SMG2 samples. (A) Red, blue and green represent biological process, cellular component and molecular function, respectively. (B) Up‐ and down‐regulated genes enriched for three ontologies, biological process, molecular function and cellular component from left to right, respectively. Red represents up‐regulated genes, and green represents down‐regulated genes. (C) The size of dot represents the number of DEGs. Rich Factor refers to the ratio between DEGs enriched in this pathway and all the annotated genes in this pathway. A large enrichment factor denotes a high degree of enrichment. The lower the *q*‐value, the more significant the enrichment of DEGs. GO, gene ontology; DEGs, differentially expressed genes; NG2, cells induced for 2 d under normal ground condition; SMG2, cells induced for 2 d under simulated microgravity

The second day (day 2) post‐induction was chosen to analyse the gene ontology (GO) of differentially expressed genes in the early stage of osteogenesis. To facilitate the analysis, 10 of the most enriched GO terms of three ontologies (biological process, cellular component and molecular function) were selected. As shown in Figure 2A, GO analysis indicated that most of the enriched DEGs were related to cell cycle and cell division in three ontologies. Upon analysis of the top enriched GO terms for the three ontologies, it was found that in almost all the terms, down‐regulated genes make up the majority (Figure 2B). In the cell cycle, 89.93% of the genes were down‐regulated, while in the chromosome in cellular component and binding in molecular function, 82.65% and 60.50% of the genes were down‐regulated, respectively. Kyoto encyclopaedia of genes and genomes enrichment (KEGG) analysis showed that these genes were involved in various metabolic pathways when cells were induced for two days (Figure 2C). Almost one tenth of DEGs were enriched in cell cycle (8.88%), and cell cycle was the most significantly enriched pathway. Among the 19 genes enriched for cell cycle, 18 genes were down‐regulated with *MCM5* (−2.48‐fold), *CCNA2* (−2.46‐fold), *CCNB1* (−3.61‐fold), *CDK1* (−5.03‐fold), *E2F1* (−3.07‐fold), *CDC25B* (−2.01‐fold) and CDC25C (−16.22‐fold) being the most prominent. Moreover, we also found that there were two GO terms related to tubulin and cytoskeleton in molecular function (Figure 2A,B).

**Figure 3:**
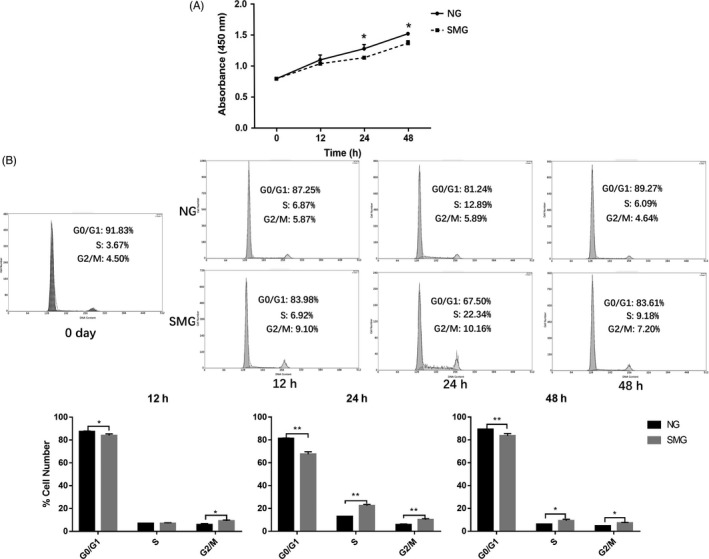
Cell proliferation and cell cycle analysis for hBMSCs induced for 0, 12, 24 and 48 h under NG and SMG. (A) Cell proliferation assay at 0, 12, 24 and 48 h. The solid line represents cells induced under normal ground condition (NG), and the dashed line represents cells induced under simulated microgravity (SMG) (n = 3). (B) The percentage of G0/G1, S and G2/M phase of cells induced for 12, 24 and 48 h under NG and SMG (n = 3), and the percentage of cells in G0/G1, S and G2/M phase determined by flow cytometry after PI staining (n = 3). Black represents cells induced under NG condition, and grey represents cells induced under SMG condition. *P < 0.05, **P < 0.01. hBMSCs, human bone marrow mesenchymal stem

To verify this result, cell proliferation, cell cycle assays and immunofluorescence of actin cytoskeleton were performed to determine the effect of microgravity. The proliferation of cells was detected at 0, 12, 24 and 48 hour of osteogenic induction. Growth curve analysis of cells revealed their capacity to proliferate under both NG and SMG, albeit with different proliferation rates (Figure 3A). The proliferation rate of cells was higher under NG compared to SMG. The cell cycle assay showed that most of cells at 0 hour were in G0/G1 phase, and few in S and G2/M phase (Figure 3B). When cells in the SMG group were induced for 12 hour, there was no change in the proportion of cells in S phase, but the number of cells in G0/G1 phase decreased while the number of cells in G2/M phase increased in comparison with the NG group. After induction for 24 hour under SMG, the percentage of cells in S and G2/M phase increased significantly in the SMG group over the NG group. Although, the percentage of S and G2/M phase reduced at 48 hour irrespective of the group, the S and G2/M phase in SMG group still accounted for a larger proportion significantly. In addition, fluorescence images of actin cytoskeleton of cells induced for 48 hour under SMG and NG conditions showed that the arrangement of cytoskeleton lost regularity under SMG and the length became shorter than that under NG condition (Figure S3).

**Figure 4:**
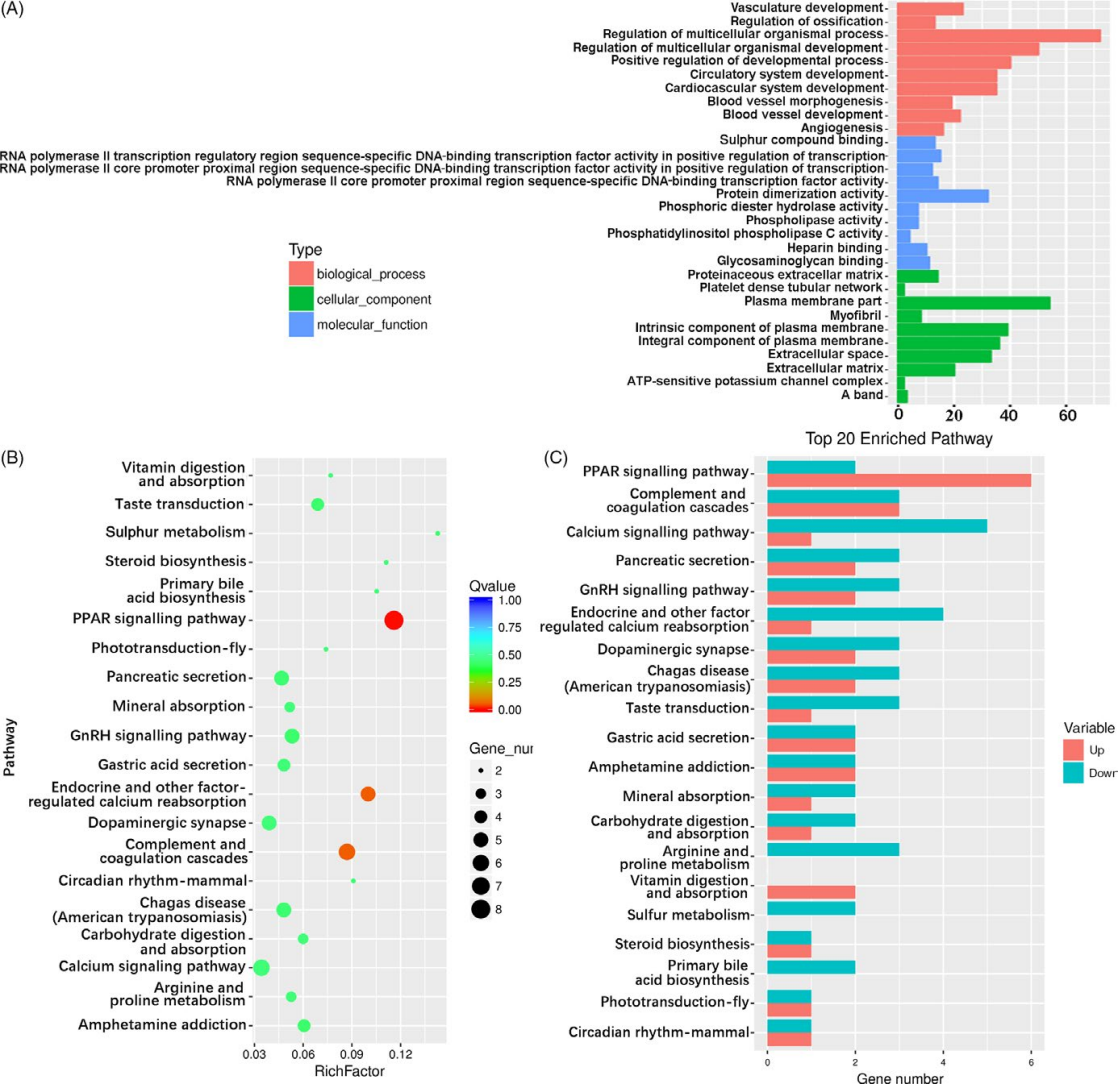
GO and pathway analysis of DEGs in NG7 vs SMG7 samples. (A) GO analysis of DEGs in three ontologies. Red, blue and green represent biological process, cellular component and molecular function, respectively. (B) Top 20 statistics of pathway enrichment for NG7 vs SMG7. The size of dot represents the number of DEGs. A large enrichment factor denotes a high degree of enrichment. The lower the *q*‐value, the more significant the enrichment of DEGs. (C) Up‐ and down‐regulated genes of top 20 enrichment pathways. Red represents up‐regulated genes, and green represents down‐regulated genes. GO, gene ontology; DEGs, differentially expressed genes; NG7, cells induced for 7 d under normal ground condition; SMG7, cells induced for 7 d under simulated microgravity

**Figure 5:**
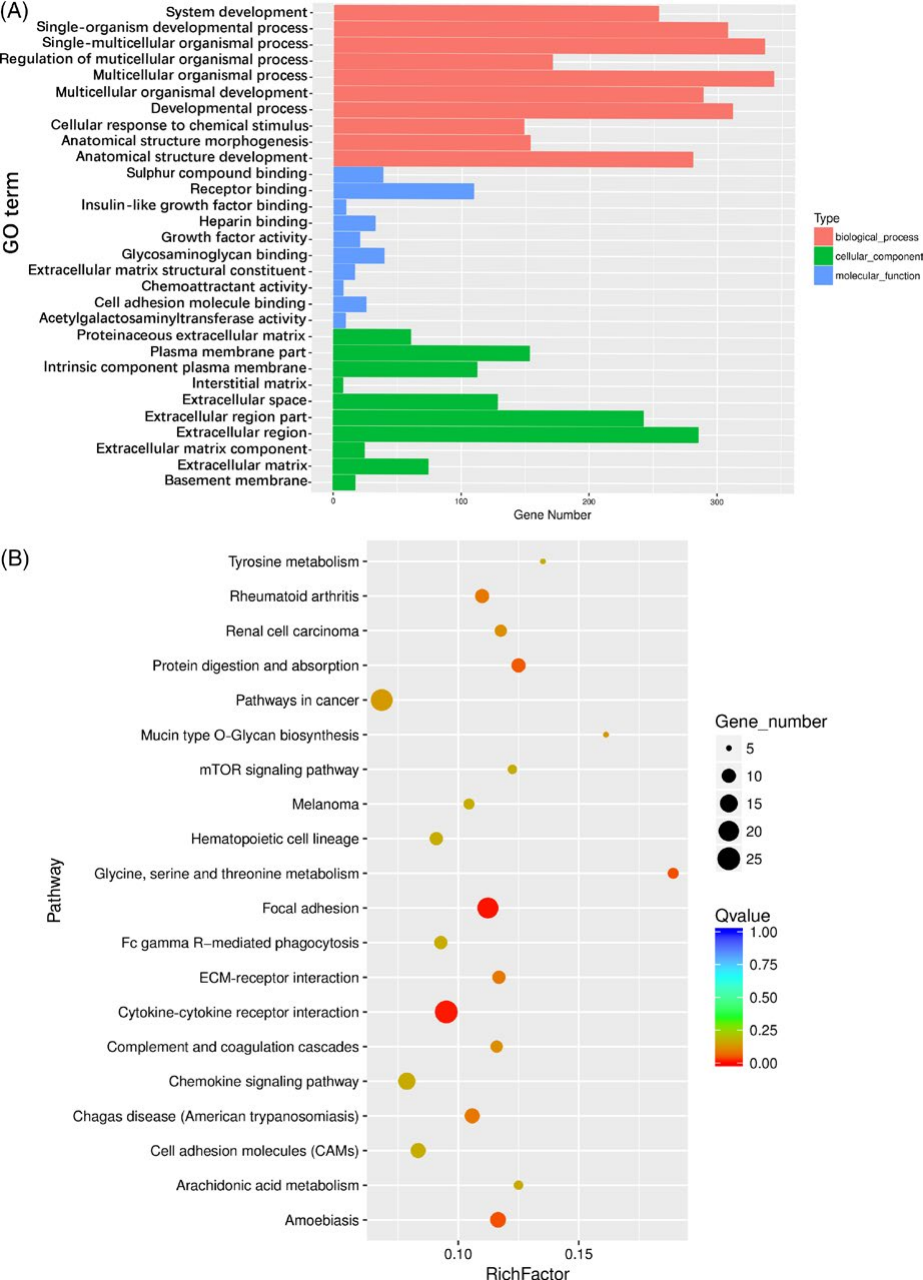
GO and pathway analysis of DEGs in NG14 vs SMG14 samples. (A) GO analysis of DEGs in three ontologies. Red, blue and green represent biological process, cellular component and molecular function, respectively. (B) Top 20 statistics of pathway enrichment for NG14 vs SMG14. The size of the dots represents the number of DEGs. Red means a smaller *q*‐value, and blue means a greater *q*‐value. A smaller *q*‐value denotes a more significant enrichment. GO, gene ontology; DEGs, differentially expressed genes; NG14, cells induced for 14 d under normal ground condition; SMG14, cells induced for 14 d under simulated microgravity

To study the middle and later stages of osteogenesis, days 7 and 14 were chosen to perform GO and KEGG analysis. In SMG7, 10 of the most enriched GO terms of three ontologies were selected (Figure 4A and Figure S4). GO analysis indicated that most of the enriched DEGs were related to multicellular organismal process. The pathway results showed that PPAR signing pathway and calcium signalling pathway were the most enriched pathways (Figure 4B). In the PPAR signalling pathway, two‐third of the genes were up‐regulated (Figure 4C); some amongst them, such as *PPARγ* (2.21‐fold), *FABP3* (4.03‐fold), *FABP4* (2.22‐fold), *PLIN1* (2.28‐fold) and *SCD* (2.06‐fold) were important for adipose differentiation. In the calcium signalling pathway, 80% of the DEGs were down‐regulated (Figure 4C). In NG14 vs SMG14 group, multicellular organismal process, receptor binding and extracellular region were the three most enriched GO terms of the three ontologies studied (Figure 5A and Figure S5). Analysis of the top 20 statistics of pathway enrichment (Figure 5B) revealed that 31.94% of the genes were enriched in cancer, cytokine‐cytokine receptor interaction and focal adhesion pathways; the cytokine‐cytokine receptor interaction was the most significant enrichment pathway. It was observed that the genes enriched in these pathways were associated with immune response, tumour progression, proliferation, differentiation and signal transduction.

Alkaline phosphatase (ALP) and Oil Red O staining assays were performed to detect osteogenic and adipogenic differentiation of hBMSCs induced with osteogenic induction medium under SMG. Oil Red O staining assay showed that on days 7 and 14 the hBMSCs under SMG tended to differentiate into adipocytes, especially at 14 days, while there was no significant change under NG condition (Figure S6A). Furthermore, with ALP assay, no significant difference was found between the NG and SMG groups on days 2, but a significant difference in the activity of ALP was detected on days 7 and 14. However, there was no significant change in ALP activity at the three time‐points within the SMG group (Figure S6B). Cell proliferation assays on days 7 and 14 were also performed to study the effect of prolonged SMG. Interestingly, enhanced proliferation of cells in SMG14 group was found (Figure S6C). However, there was no significant difference in SMG7 and NG7.

### 3.4 Validation of DEGs by qRT‐PCR

Quantitative real‐time PCR (qRT‐PCR) was performed to validate the differentially expressed genes involved in the RNA‐seq data (Figure S7). Thirteen genes were selected from the differentially expressed genes of different time periods. Of these genes, five were involved in cell cycle, four were associated with osteogenesis and four were encoded proteins with adipogenesis. In 2 days, genes related to the cell cycle (*CDKN3*,* MCM5*,* CCNB1*,* CDK1* and *CDC20*) were significantly down‐regulated, while the genes specific for osteogenic differentiation, *RunX2*,* ALPL, BMP2* and *COL1A1* were down‐regulated. Of four genes specific for adipogenic differentiation, *PPARγ*,* CEBPA*,* CEBPB* and *CFD* were up‐regulated. In 7 days, although the genes specific for cell cycle were up‐regulated, no significant difference between NG and SMG groups were observed. The genes specific for osteogenic differentiation were significantly down‐regulated, and all the genes specific for adipogenic differentiation were significantly up‐regulated. In 14 days, we found the same trend as 7 days in the genes specific for differentiation, but the cell cycle‐related genes *MCM5*,* CCNB1* and *CDC20* were significant up‐regulated. The results showed that the expression patterns of these thirteen genes were highly in agreement with the RNA‐seq results.

## 4 DISCUSSION

In the present study, whole transcriptome analysis revealed that SMG affected many biological processes of hBMSCs. Not only tissue‐specific genes but also genes related to proliferation and differentiation were affected. On day 2, hBMSCs cultured under SMG exhibited down‐regulation of the genes related to cell cycle, such as *MCM5*, CCNA2, *CCNB1*,* CDK1*,* E2F1*,* CDC25B* and *CDC25C*.^16^, ^17^, ^18^, ^19^, ^20^, ^21^, ^22^ It has been reported that microgravity changes the distribution of the cell cycle phase in many mammalian cells, such as primitive human haematopoietic progenitor cells^23^ and endothelial cells.^24^ In addition, several studies have shown that microgravity has a major effect on the cytoskeleton,^25^, ^26^ and changes in cytoskeleton may play an important role in the cell cycle. Alterations in cytoskeletal structure can block cell growth in G2/M (inhibition of microtubule polymerization during G2/M phase).^27^ In our experiments, we observed that the cytoskeleton of the cells induced for 48 hour under SMG was disordered and the length of the cytoskeleton was shorter than that under NG condition. The decrease in the above genes and the change of the cytoskeleton resulted in the arrest of the cell cycle in S phase and G2/M transitions, and the interaction of these changes led to an increase in the proportion of cells in S and G2/M phases. These results were different from the previous report^28^ showing that the cell cycle was arrested in G0/G1 phase. However, our results were consistent with another study showing that modelled μ‐g induced alterations in cell cycle kinetics characterized by prolonged S phase and reduced cyclin A expression.^23^ The previous study showed that continuous inhibition of genes cyclin A and *CDC2* inhibits the cell exit from S phase.^29^ In our results, genes of *CCNA2* (cyclin A, −2.46‐fold) and *CDK1* (*CDC2*, −5.03‐fold) were significantly down‐regulated, which maybe contributed to the arrest of hBMSCs in S phase. Damm et  al^30^ also reported that culturing cells on the RPM for 6 and 12 hour resulted in their depletion from the G0/G1 phases, concomitant with their accumulation in the G2/M phase. In our RNA‐seq results, some check point genes, such as *CCNB1* (−3.61‐fold) which plays a role in the regulation of G2/M checkpoint of the cell cycle,^18^ *CDC25B* (−2.01‐fold) that is a dual specificity phosphatase which accumulates during the late S and early G2 phases of the cell cycle and is essential for the G2/M transition^21^ and *CDC25C* which plays a key role in G2/M phase transition,^31^ were significantly down‐regulated. The down‐regulation of these check point genes may also be contributed to the arrest of hBMSCs in G2/M phase. In addition, the proliferation inhibition of hBMSCs may be resulted from various factors, such as the osteogenic differentiation, SMG or their combination. In this study, however, it is inferred that the lower proliferation rate of hBMSCs under SMG in comparison with that under normal gravity should be resulted from SMG because these experiments were performed under the same osteogenic induction condition. The results of cell cycle analysis indicated that hBMSCs were mainly arrested in the S and G2/M phases under SMG, and our proliferation experiment showed that SMG decreased the proliferation potential of cells in the early stage of osteogenic induction.

Gene ontology (GO) and KEGG pathway analysis showed that the differentiation of hBMSCs was mainly affected by SMG in the middle stage of induction (day 7). Many studies have reported that microgravity inhibited osteogenic differentiation and enhanced adipogenic differentiation.^32^, ^33^ In agreement with these reports, KEGG pathway enrichment in our study showed that the PPAR signalling pathway and calcium signalling pathway were the most enriched pathways with two‐thirds of the DEGs in PPAR signalling pathway being up‐regulated, while 80% of the DEGs in calcium signalling pathway being down‐regulated. From previous reports, we know that PPAR‐γ, a well‐known adipogenic marker, powerfully induces adipogenesis at the morphological and molecular levels in response to a number of PPAR‐γ activators.^34^ *CEBPA* (2.52‐fold) has also been showed to have roles in differentiation and lipogenesis.^35^ *PPAR‐γ* is essential for the differentiation of adipocytes in vivo and in vitro, and *CEBPA* has been shown to induce adipocyte differentiation through transactivation of *PPAR‐γ*.^36^ Up‐regulation of these genes promotes adipogenic differentiation of hBMSCs under SMG. Calcium signalling pathway plays an important role in osteogenic differentiation.^37^, ^38^ The expression of osteogenic genes in hBMSCs was highly increased with high extracellular Ca^2+^ concentration in the in vitro environment,^39^ and Ca^2+^ influx through the transient receptor potential melastatin type 7 (TRPM7) further triggered Ca^2+^ release from the inositol trisphosphate receptor type 2 on the endoplasmic reticulum and promoted osteogenesis.^40^ In addition to inhibiting the osteogenesis‐specific genes, inhibition of calcium signalling pathway is also responsible for SMG inhibition of osteogenic differentiation. In the later stages of induction (day 14), GO enrichment analysis showed that SMG mainly affected the multicellular organism process and developmental process. Through pathway enrichment, we found that cytokine‐cytokine receptor interaction and pathways in cancer were the most enriched pathways. From these DEGs, we found that many genes associated with growth factors, cytokines, tumorigenesis and angiogenesis were up‐regulated, while the genes associated with apoptosis, inflammation and signal transduction were down‐regulated. Previous reports have suggested that prolonged spaceflight has a negative impact on the immune system resulting in hypoplasia of lymphoid organs and alterations in mitogen‐induced blastogenesis.^41^ Under SMG, a significant loss of antigen‐specific cytotoxic T lymphocyte activity was observed, suggesting the negative effect of SMG on cell‐mediated immunity.^42^ Moreover, microgravity has adverse effects on tumour cells. Although cancer stem cells are committed to selective differentiation when cultured in microgravity, a study has provided evidence of significant apoptosis under this condition.^43^ Another study reported that some tumours seem to be much less aggressive in the microgravity environment of space compared to Earth.^44^ Nevertheless, SMG may have altered the tumour cell characteristics and enhanced the invasive property. Therefore, it is possible that the microgravity analogue culture environment may have selected highly tumorigenic cells for survival.^45^ Our RNA‐seq results are more inclined to support this conclusion.

To confirm the effect of simulated microgravity on osteogenic differentiation and adipogenic differentiation derived from RNA‐seq data, we performed assays of ALP activity and Oil Red O staining. ALP activity, as a marker of osteogenic differentiation, reflects bone formation and/or mineralization.^11^ We found that ALP activity at 7 and 14 days was significantly increased compared to that at 2 days in NG group, but no obvious change in SMG group. These results indicated that SMG inhibited osteogenic differentiation of hBMSCs. For analysis of adipogenic potential of hBMSCs under osteogenic induction, we found the significantly positive staining of Oil Red O in SMG group compared to those in NG group, especially at 14 days, which shows a tendency of adipogenic differentiation even under osteogenic induction. These results are consistent with the conclusion of RNA‐seq data.

In conclusion, SMG inhibits the proliferation of cells in the early stage of osteogenesis of hBMSCs while in the middle stage, it inhibits the differentiation towards osteoblasts and promotes adipogenesis. It may select highly tumorigenic cells for survival under prolonged SMG. We believe that the data provided in this study may clarify some of the physiological changes, such as bone loss and immune system dysfunction occurring during spaceflight, and in favour of a better understanding of the mechanism of these changes.

## CONFLICT OF INTERESTS

Authors indicate no potential conflict of interests.

## AUTHOR CONTRIBUTIONS

J.F.W. and P.C. designed the study. L.L. conducted the study. L.L. and C.Z. collected the data. L.L. and C.Z. analysed the data. L.L., C.Z., J.L.C. and F.F.H. interpreted the data. L.L. drafted the manuscript. C.Z., J.F.W. and P.C. revised the manuscript content. L.L., C.Z., J.L.C., F.F.H. J.F.W. and P.C. approved the final version of the manuscript. L.L. takes responsibility for the integrity of the data analysis.
