# Supplementary material for: Effects of simulated microgravity on the expression profiles of RNA during osteogenic differentiation of human bone marrow mesenchymal stem cells
Source: Cell Prolif. 2018 Nov 5;52(2):e12539. doi: 10.1111/cpr.12539 (PMC6496301; doi:10.1111/cpr.12539)
Supplement: Supplementary file 1 [file CPR-52-e12539-s001.docx]

**Effects of simulated microgravity on the expression profiles of RNA during osteogenic differentiation of** **human bone marrow mesenchymal stem cells**

Effects of simulated microgravity on stem cells

Liang Li ^1^, Cui Zhang ^1^, Jian-ling Chen ^1^, Fan-fan Hong ^1^, Ping Chen^2^*, Jin-fu Wang ^1^*

1. Institute of Cell and Development Biology, College of Life Sciences, Zijingang Campus, Zhejiang University, Hangzhou, Zhejiang 310058, P. R. China

2. Departments of Cell Biology and Otolaryngology, Emory University School of Medicine, Atlanta, Georgia 30322, USA.

* Corresponding author: Jin-Fu Wang, College of Life Sciences, Zijingang Campus, Zhejiang University, number 866 of Yuhangtang road, West Lake Dist, Hangzhou, Zhejiang Province, P. R. China. E-mail: [wjfu@zju.edu.cn](mailto:wjfu@zju.edu.cn). Ping Chen, Departments of Cell Biology and Otolaryngology, Emory University School of Medicine, Atlanta, Georgia 30322, USA. E-mail: [pchen2@emory.edu](mailto:pchen2@emory.edu)


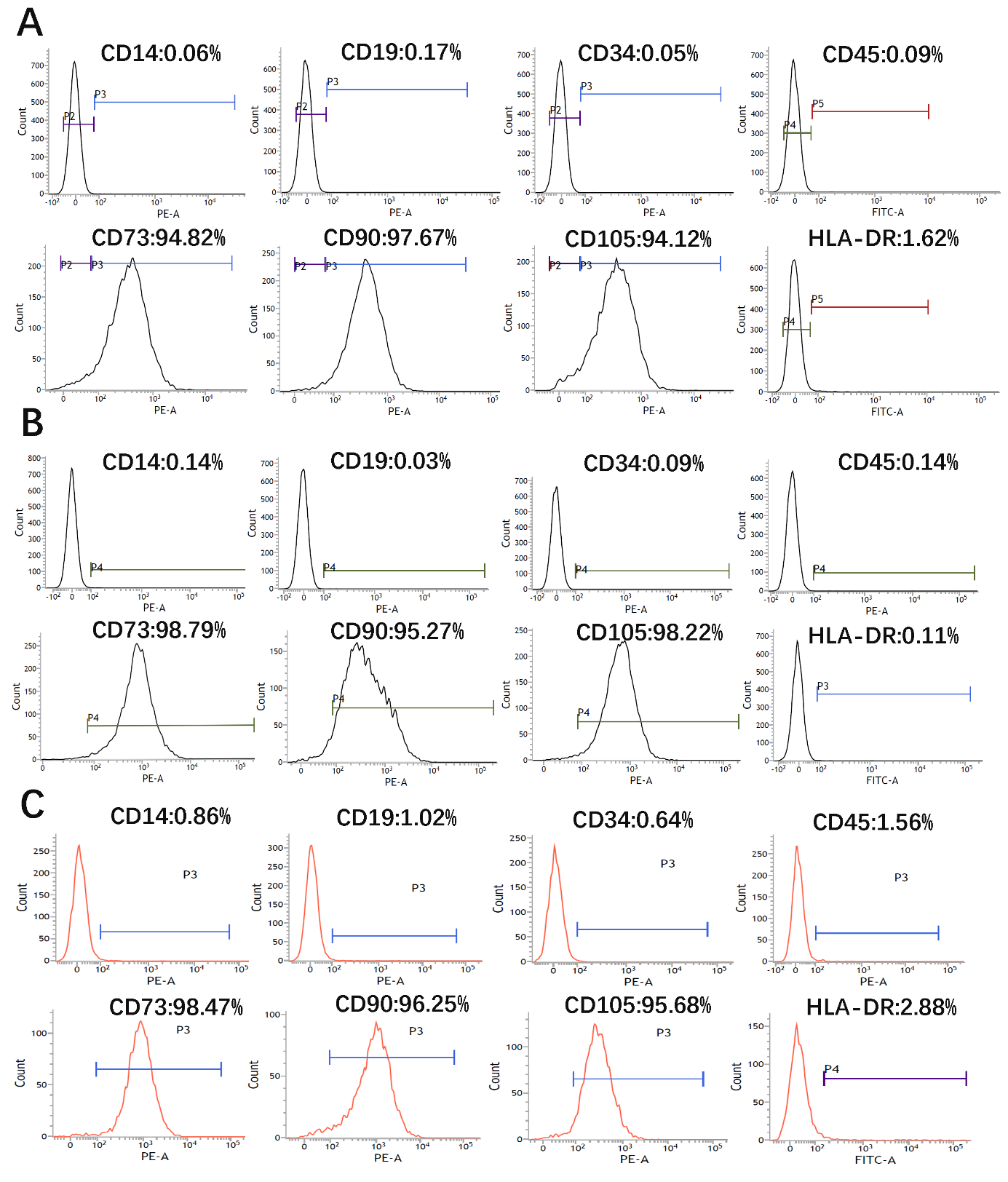


**Fig S1.** **Analysis of surface markers of hBMSCs.** (A) Flow cytometric analysis of surface markers in hBMSCs isolated from 23-year-old man showed positive for CD29, CD73, CD90, and CD105 and negative for CD34, CD19, CD45, CD14, and HLA-DR. (B) Flow cytometric analysis of surface markers in hBMSCs isolated from 19-year-old woman showed positive for CD29, CD73, CD90, and CD105 and negative for CD34, CD19, CD45, CD14, and HLA-DR. (C) Flow cytometric analysis of surface markers in hBMSCs isolated from 34-year-old man showed positive for CD73, CD90, and CD105 and negative for CD34, CD19, CD45, CD14, and HLA-DR. hBMSCs, human bone marrow mesenchymal stem cells.


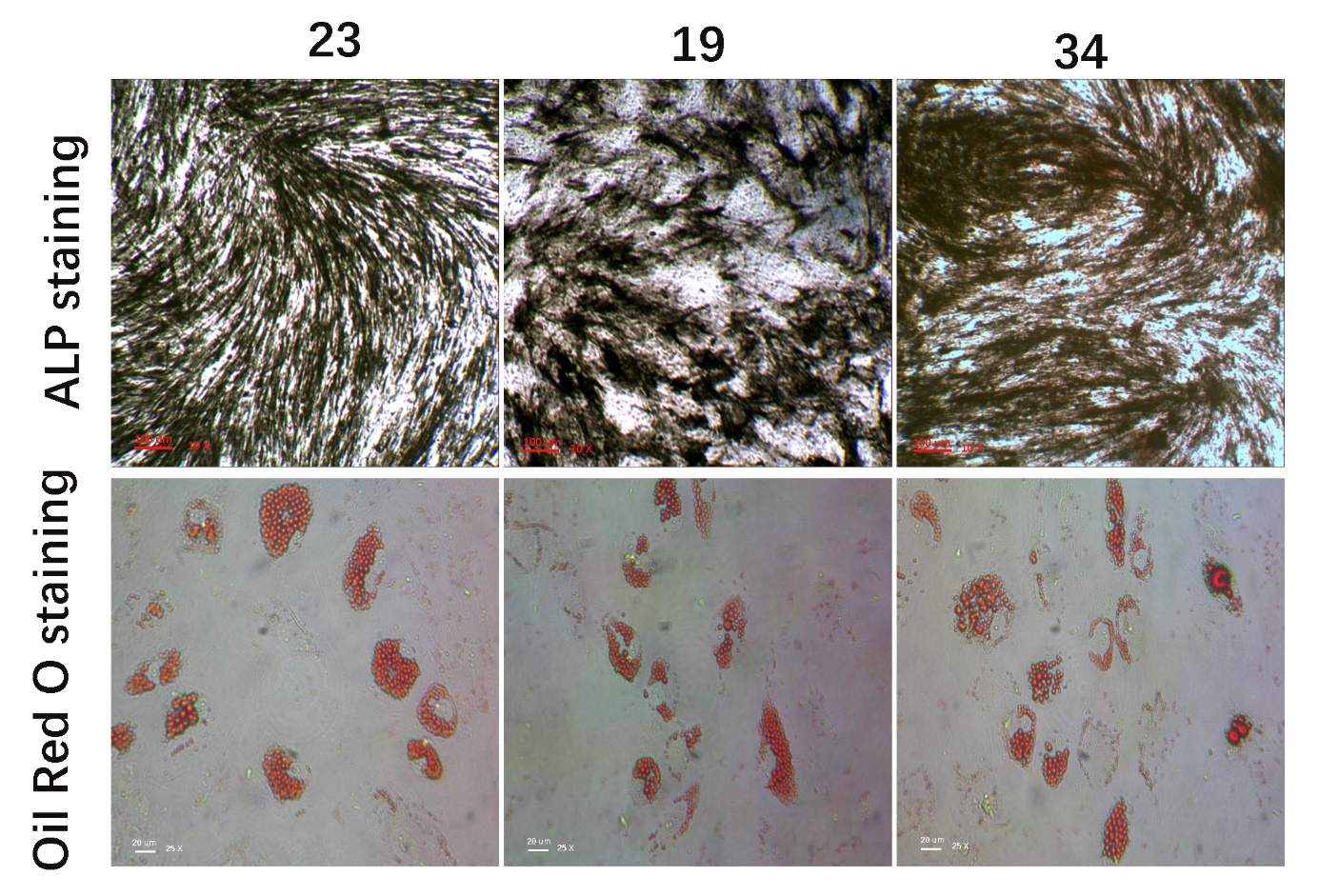


**Fig S2. Analysis of differentiation potentials of hBMSCs.** The osteogenic and adipogenic potentials of hBMSCs isolated from 23-year-old man, 19-year-old woman and 34-year-old woman were evaluated by ALP and Oil Red O staining. ALP, alkaline phosphatase; hBMSCs, human bone marrow mesenchymal stem cells.


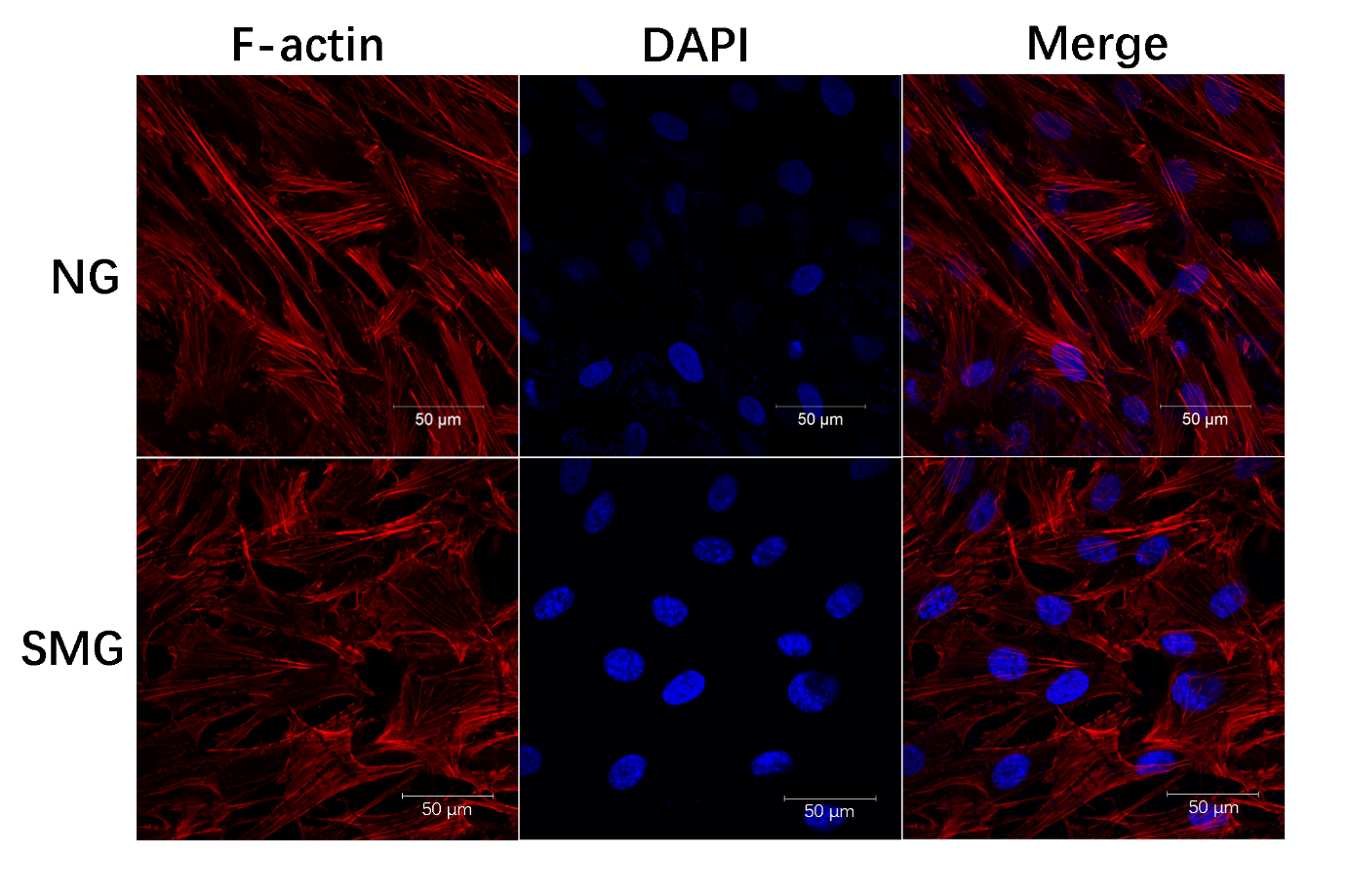


**Fig S3. Fluorescence images of the actin cytoskeleton (red) and nucleus (blue) of hBMSCs cultured for 48 h in NG and SMG.** hBMSCs, human bone marrow mesenchymal stem cells; NG, normal ground condition; SMG, simulated microgravity.


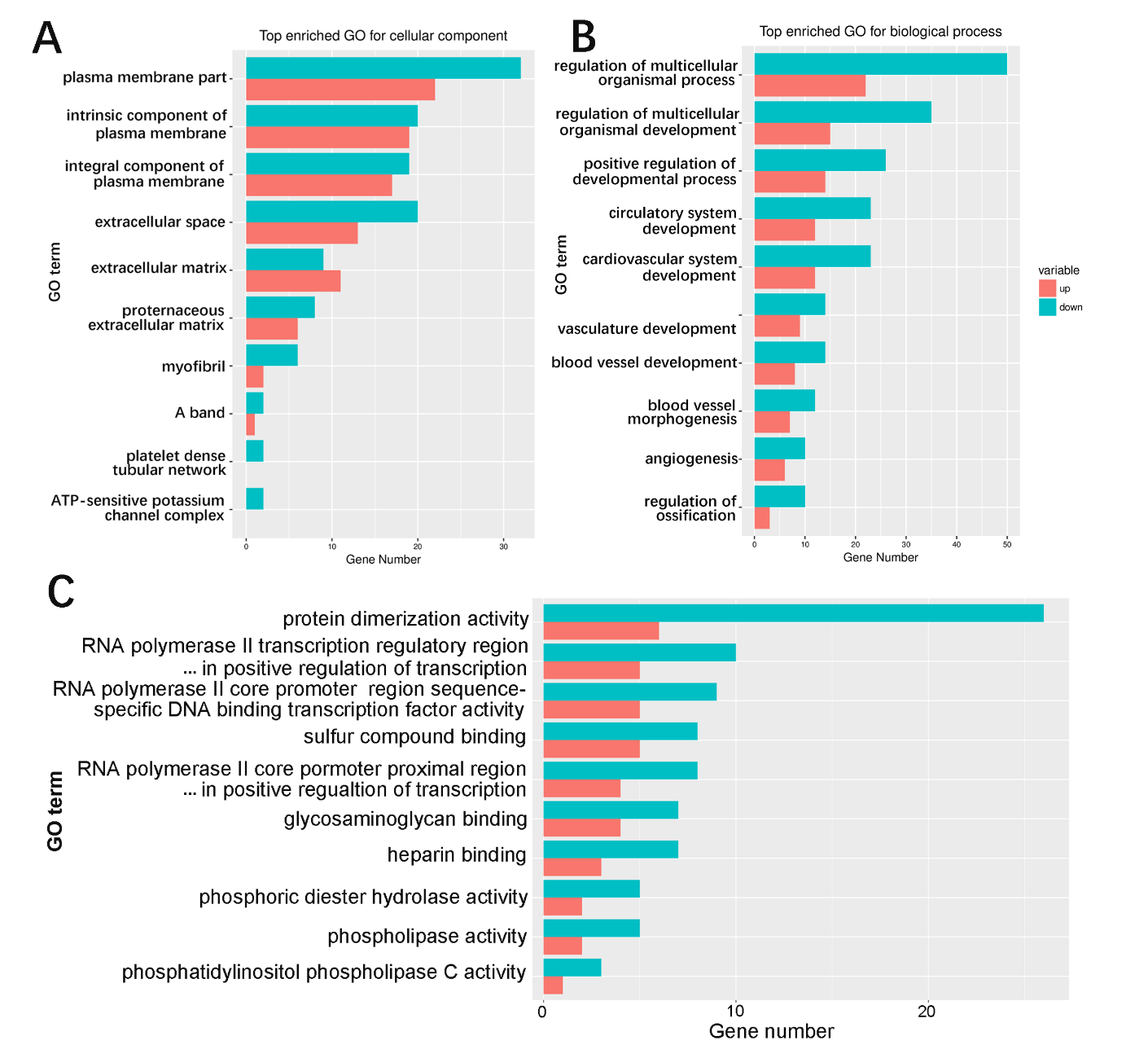


**Fig S4. Up- and down-regulated genes in NG7 vs SMG7 samples.** (A) Up- and down-regulated genes of top enriched GO terms for cellular component. (B) Up- and down-regulated genes of top enriched GO terms for biological process. (C) Up- and down-regulated genes of top enriched GO terms for molecular function. GO, gene ontology.


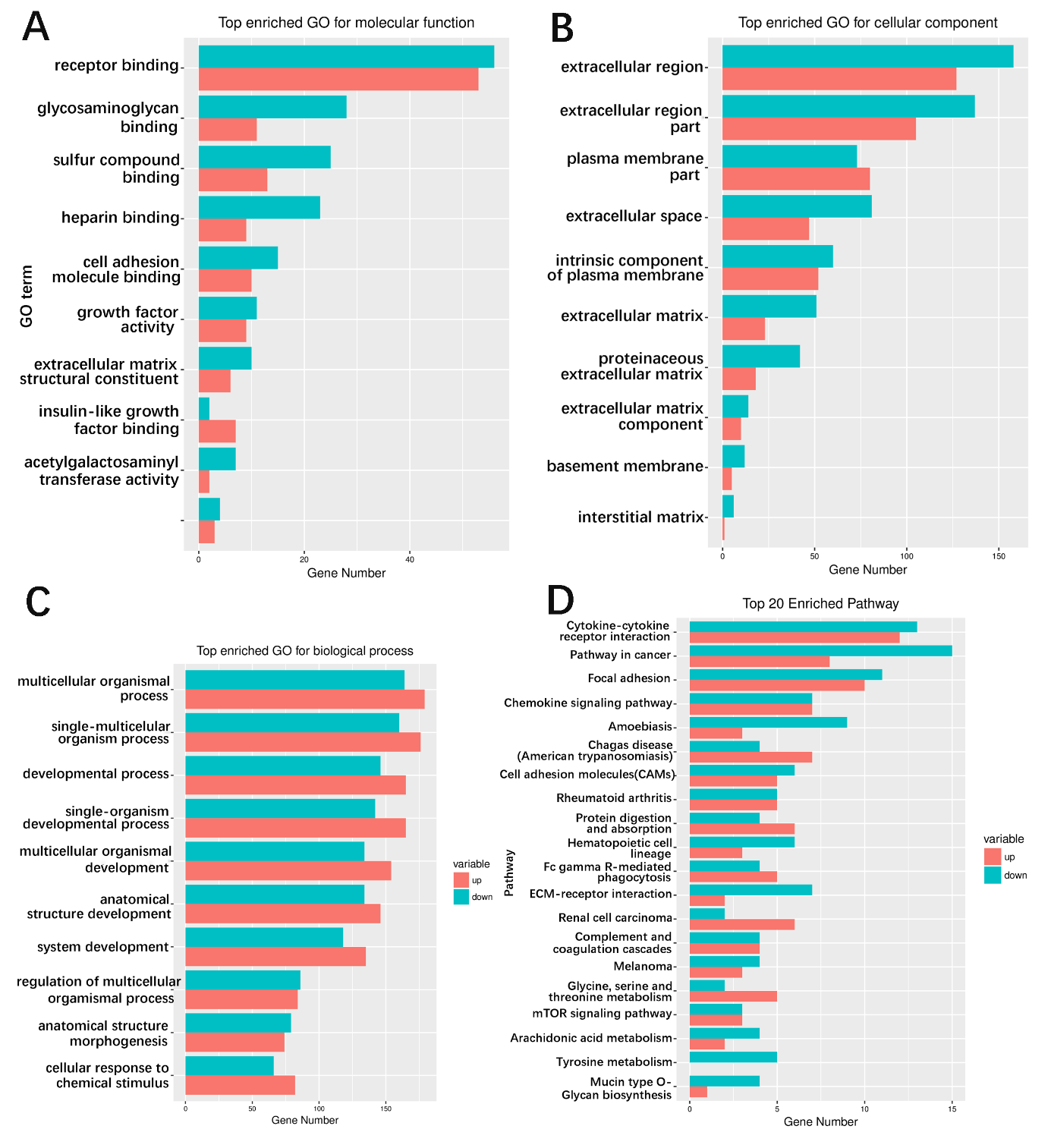


**Fig S5. Up- and down-regulated genes in NG14 vs SMG14 samples.** (A) Up- and down-regulated genes of top enriched GO terms for molecular function. (B) Up- and down-regulated genes of top enriched GO terms for cellular component. (C) Up- and down-regulated genes of top enriched GO terms for biological process. (D) Up- and down-regulated genes of top enriched pathway. GO, gene ontology.


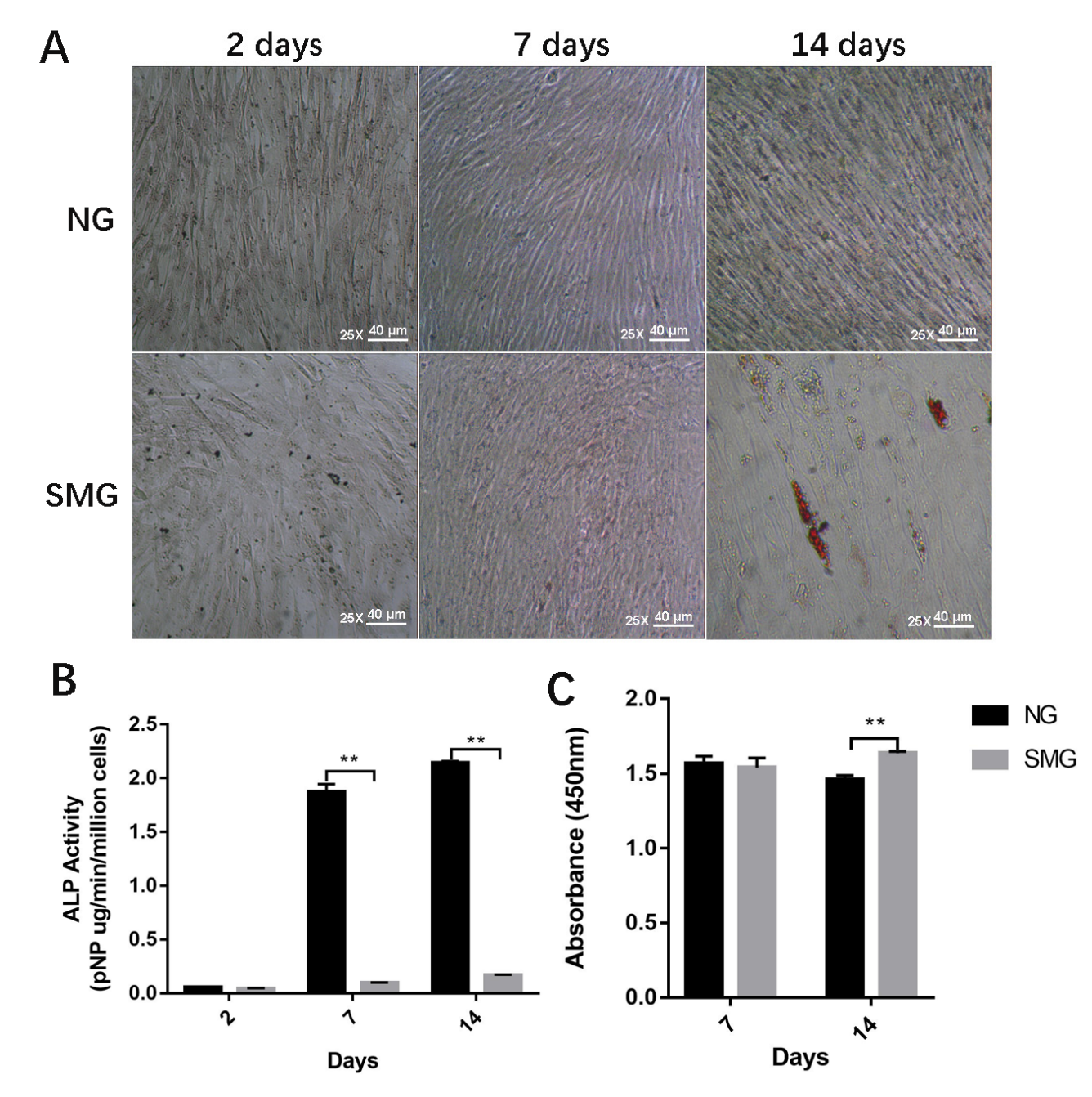


**Fig S6. Differentiation and proliferation of hBMSCs in the middle and later stages of osteogenesis under SMG.** (A) Oil Red O staining of hBMSCs after 2, 7, and 14 days of osteogenic induction under NG and SMG. (B) The relative activity of alkaline phosphatase (ALP) after exposure to SMG and NG for different time points (n=3). (C) The proliferation of hBMSCs induced for 7 and 14 days under NG and SMG. Black represents cells induced under NG and gray represents cells induced under SMG (n=3). ** p < 0.01. NG, normal ground condition; SMG, simulated microgravity; hBMSCs, human bone marrow mesenchymal stem cells.


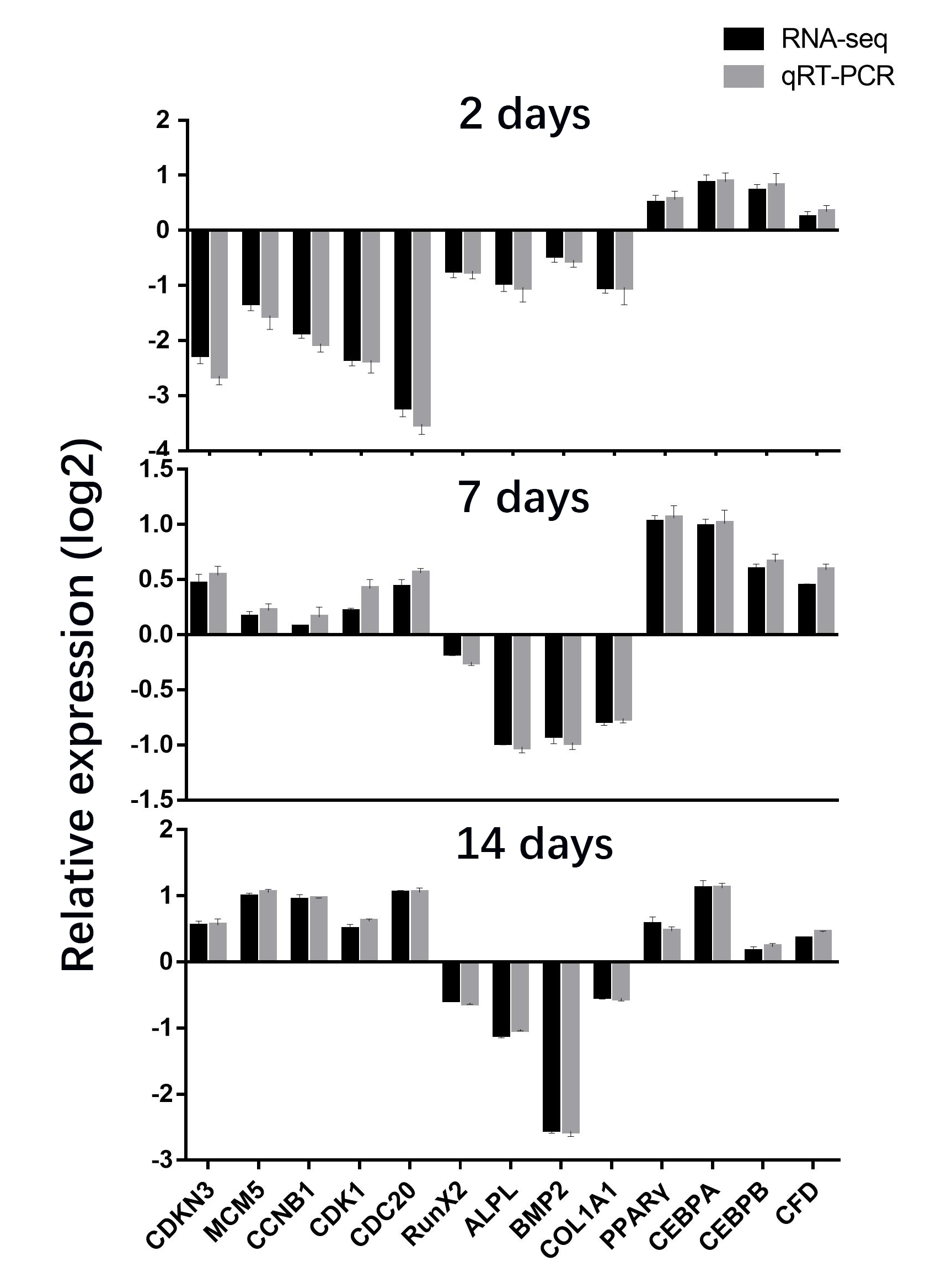


**Fig S7. RNA-seq data validation by quantitative real-time PCR (qRT-PCR).** (A) RNA-seq and qRT-PCR data of 2 days sample. (B) RNA-seq and qRT-PCR data of 7 days sample. (C) RNA-seq and qRT-PCR data of 14 days sample. qRT-PCR was used to confirm the differential expression of thirteen genes of RNA-seq: *CDKN3*, *MCM5*, *CCNB1*, *CDK1*, *CDC20*, *RunX2*, *ALPL*, *BMP2*, *COL1A1*, *PPARγ*, *CEBPA*, *CEBPB*, and *CFD*. Fold changes were determined by the 2^−ΔΔCT^ relative quantitation method using 18S rRNA as the endogenous control. RNA-seq and qRT-PCR results are represented in black and gray, respectively.
