# Supplementary material for: Effects of simulated microgravity on the expression profiles of RNA during osteogenic differentiation of human bone marrow mesenchymal stem cells
Source: Cell Prolif. 2018 Nov 5;52(2):e12539. doi: 10.1111/cpr.12539 (PMC6496301; doi:10.1111/cpr.12539)
Supplement: Supplementary file 2 [file CPR-52-e12539-s002.docx]

Table S1. Primer sequences used for qPCR analysis

| Gene name | Forward primer (5’-3’) | Reverse primer (5’-3’) | Amplicon length | Annotation |
| --- | --- | --- | --- | --- |
| CDKN3 | ATGAAGCCGCCCAGTTCAAT | TCACTCGTGACAAAGATAGCCA | 112 | cyclin-dependent kinase inhibitor 3 |
| MCM5 | GGAAGTGCAACACAGATCAGG | AGGGACGACCTTGTCACACA | 194 | minichromosome maintenance complex component 5 |
| CCNB1 | AGCTGCTGCCTGGTGAAGAG | GCCATGTTGATCTTCGCCTTA | 91 | cyclin B1 |
| CDK1 | GGATGTGCTTATGCAGGATTCC | CATGTACTGACCAGGAGGGATAG | 100 | cyclin-dependent kinase 1 |
| CDC20 | GCCCACCAAGAAGGAACATC | TTTTCCACTGAGCCGAAGGA | 91 | cell division cycle 20 homolog |
| RunX2 | CCAACCCACGAATGCACTATC | TAGTGAGTGGTGGCGGACATAC | 91 | runt related transcription factor 2 |
| ALPL | AACATCAGGGACATTGACGTG | GTATCTCGGTTTGAAGCTCTTCC | 159 | alkaline phosphatase, liver/bone/kidney |
| BMP2 | ACTACCAGAAACGAGTGGGAA | GCATCTGTTCTCGGAAAACCT | 113 | bone morphogenetic protein 2 |
| COL1A1 | GTGCGATGACGTGATCTGTGA | CGGTGGTTTCTTGGTCGGT | 119 | collagen type I alpha 1 chain |
| PPARγ | ACCAAAGTGCAATCAAAGTGGA | ATGAGGGAGTTGGAAGGCTCT | 199 | peroxisome proliferator activated receptor gamma |
| CEBPA | AAGCACGATCAGTCCATCCC | GGCACAGAGGCCAGATACAA | 100 | CCAAT enhancer binding protein alpha |
| CEBPB | CTTCAGCCCGTACCTGGAG | GGAGAGGAAGTCGTGGTGC | 136 | CCAAT/enhancer binding protein beta |
| CFD | GACACCATCGACCACGACC | GCCACGTCGCAGAGAGTTC | 128 | complement factor D |
