# Supplementary material for: Effects of simulated microgravity on the expression profiles of RNA during osteogenic differentiation of human bone marrow mesenchymal stem cells
Source: Cell Prolif. 2018 Nov 5;52(2):e12539. doi: 10.1111/cpr.12539 (PMC6496301; doi:10.1111/cpr.12539)
Supplement: Supplementary file 3 [file CPR-52-e12539-s003.docx]

Table S2. Summary of data from RNA-seq for 7 samples under NG and SMG conditions

|  | d0 | SMG2 | NG2 | SMG7 | NG7 | SMG14 | NG14 |
| --- | --- | --- | --- | --- | --- | --- | --- |
| Total raw reads | 44392050 | 46012382 | 51169266 | 56579784 | 44873566 | 54567246 | 58549658 |
| Total clean reads | 44045212 | 45635468 | 50730070 | 56305892 | 44462804 | 54034438 | 58192318 |
| Clean GC (%) | 52.13 | 52.11 | 52.16 | 51.62 | 51.56 | 52.34 | 51.95 |
| PE mapped reads (%) | 91.06 | 91.14 | 91.03 | 90.6 | 90.98 | 90.19 | 89.9 |
| Adapter (%) | 0.78 | 0.82 | 0.86 | 0.48 | 0.91 | 0.97 | 0.61 |
| Q30 reads (%) | 90.95 | 91.03 | 90.99 | 90.99 | 91.09 | 90.84 | 90.65 |
| Exon (%) | 88.91 | 90.22 | 90.69 | 91.65 | 92.54 | 92.73 | 90.91 |
| Intron (%) | 8.29 | 7.21 | 6.96 | 6.66 | 6.14 | 5.84 | 6.99 |
| Intergenic (%) | 2.8 | 2.57 | 2.35 | 1.69 | 1.33 | 1.43 | 2.1 |

NG, normal ground condition; SMG, simulated microgravity; d0, cells induced for 0 day; NG2, cells induced for 2 days under normal ground condition; SMG2, cells induced for 2 days under simulated microgravity; NG7, cells induced for 7 days under normal ground condition; SMG7, cells induced for 7 days under simulated microgravity; NG14, cells induced for 14 days under normal ground condition; SMG14, cells induced for 14 days under simulated microgravity
