# Supplementary material for: Effects of simulated microgravity on the expression profiles of RNA during osteogenic differentiation of human bone marrow mesenchymal stem cells
Source: Cell Prolif. 2018 Nov 5;52(2):e12539. doi: 10.1111/cpr.12539 (PMC6496301; doi:10.1111/cpr.12539)
Supplement: Supplementary file 4 [file CPR-52-e12539-s004.docx]

Table S3. DEGs included in the eight main clusters

| Cluster 1 | | |
| --- | --- | --- |
| UP_KEYWORDS | Gene Number | Gene |
| Disulfide bond | 4 | CXCL10, DCC, ST8SIA2, NTF3 |
| Signal | 4 | CXCL10, DCC, ST8SIA2, NTF3 |

| Cluster 2 | | |
| --- | --- | --- |
| UP_KEYWORDS | Gene Number | Gene |
| Calmodulin-binding | 5 | ADCY1, CAMK1G, CAMK2A, CAMK4, SYT7 |
| Developmental protein | 10 | SOX11, VANGL2, CCIN, EREG, HAND2, INA, MAB21L1, MAB21L2, NGEF, SHISA2 |
| Serine/threonine-protein kinase | 6 | CAMK1G, CAMK2A, CAMK4, MAP4K1, RIPK4, RPS6KL1 |
| Intermediate filament | 3 | INA, KRT15, KRT81 |
| Kinase | 7 | EPHB1, CAMK1G, CAMK2A, CAMK4, MAP4K1, RIPK4, RPS6KL1 |
| Neurogenesis | 4 | EPHB1, SOX11, INA, NGEF |
| Differentiation | 6 | SOX11, CCIN, EREG, HAND2, INA, NGEF |
| Cytoplasmic vesicle | 5 | ATP8A1, CADPS, NPTX1, NTS, SYT7 |
| Glycoprotein | 22 | EPHB1, F2RL1, FCRLB, ST8SIA5, ACPP, ADCY1, CAMK4, CPXM1, CREG2, C3orf80, F2RL2, COL22A1, C3AR1, EREG, ELFN1, IL13RA2, INA, NTN1, NPTX1, NTSR1, TNXB, THBD |

| Cluster 4 | | |
| --- | --- | --- |
| UP_KEYWORDS | Gene Number | Gene |
| Glycoprotein | 50 | HTR2A, ADAMDEC1, ADAM28, BPIFB4, CD1D, FCRL6, LYPD3, OCA2, RSPO1, SPARCL1, TRDC, WFDC2, XPNPEP2, ADGRD1, ADGRF4, ADIPOQ, ANGPT4, ANO2, ACKR1, CNR1, CA9, CRTAC1, COL4A4, C6, DCT, EFNA1, GPC3, HS3ST2, INHBB, INSRR, LAIR1, MSLN, ORM1, PILRA, PON3, KCNC3, PSG4, PCSK2, P2RX5, RXFP1, SCARA5, SERPINA1, SHISA9, SLC6A1, TFR2, TF, TREM1, TYRP1, VEGFD, ZG16B |
| Disulfide bond | 39 | HTR2A, ADAMDEC1, ADAM28, BPIFB4, CCL20, CD1D, FCRL6, RSPO1, SPARCL1, TRDC, WFDC2, ADIPOQ, ANGPT4, ACKR1, CA9, CRTAC1, COL4A4, C6, EFNA1, HS3ST2, INHBB, INSRR, LAIR1, LDLRAD4, MSLN, ORM1, PON3, PSG4, PCSK2, P2RX5, RXFP1, SCARA5, SFRP5, SLC6A1, SYT6, TFR2, TF, TREM1, VEGFD |
| Signal | 43 | ADAMDEC1, ADAM28, BPIFB4, CCL20, CD1D, FXYD1, FCRL6, LYPD3, RSPO1, SAA2-SAA4, SPARCL1, WFDC2, XPNPEP2, ADGRD1, ADGRF4, ADIPOQ, ANGPT4, CA9, CRTAC1, COL4A4, C6, DCT, EFNA1, GPC3, INHBB, INSRR, LAIR1, MSLN, ORM1, PILRA, PON3, KCND3, PSG4, PCSK2, RXFP1, SFRP5, SERPINA1, SHISA9, TF, TREM1, TYRP1, VEGFD, ZG16B |
| Secreted | 26 | ADAMDEC1, ADAM28, BPIFB4, CCL20, RSPO1, SPARCL1, WFDC2, ADIPOQ, ANGPT4, CRTAC1, COL4A4, C6, EFNA1, GPC3, INHBB, MSLN, ORM1, PILRA, PON3, PSG4, SFRP5, SERPINA1, TF, TREM1, VEGFD, ZG16B |
| GPI-anchor | 5 | LYPD3, XPNPEP2, EFNA1, GPC3, MSLN |
| Lipoprotein | 11 | FXYD1, HCK, LYPD3, XPNPEP2, CABP1, CNR1, EFNA1, GPC3, MSLN, RHOH, RGS16 |
| Cell membrane | 26 | HTR2A, ADAM28, CD1D, HCK, LYPD3, XPNPEP2, ADGRD1, ANO2, CABP1, CNR1, CA9, EFNA1, GPC3, LAIR1, MSLN, PILRA, KCNC3, KCND3, RHOH, RXFP1, SCARA5, SHISA9, SLC6A1, SYT6, TFR2, TREM1 |
| Cleavage on pair of basic residues | 6 | ADAM28, INHBB, INSRR, MSLN, PCSK2, VEGFD" |
| Angiogenesis | 4 | ANGPT4, EFNA1, HIF3A, VEGFD |
| Melanin biosynthesis | 2 | DCT, TYRP1 |
| Copper | 3 | MT1H, MT3, TYRP1 |
| Receptor | 14 | HTR2A, TRDC, ADGRD1, ADGRF4, ACKR1, CNR1, INSRR, LAIR1, PILRA, P2RX5, RXFP1, SCARA5, TFR2, TREM1 |
| Metal-thiolate cluster | 2 | MT1H, MT3 |
| Metal-binding | 25 | ADAMDEC1, ADAM28, ADAP1, PDZRN4, SPARCL1, XPNPEP2, CABP1, CA9, CYP4B1, DCT, EGLN3, MKRN3, MT1H, MT3, NEBL, PON3, PDE4C, KCND3, RXFP1, SLC6A1, SYT6, TF, TRIM29, TYRP1, ZNF385B |
| Membrane | 45 | HTR2A, ADAM28, CD1D, FXYD1, FCRL6, HCK, LYPD3, OCA2, TRDC, XPNPEP2, AP3B2, ADGRD1, ADGRF4, ANO2, ACKR1, ATG9B, CABP1, CNR1, CA9, CYP4B1, DCT, EFNA1, GPC3, HS3ST2, INSRR, LAIR1, LDLRAD4, MSLN, PILRA, KCNC3, KCND3, P2RX5, RHOH, RGS16, RXFP1, SCARA5, SHISA9, SDR42E1, SLC6A1, SYT6, TFR2, TMEM176A, TMEM176B, TREM1, TYRP1 |
| Hydroxylation | 3 | ADIPOQ, COL4A4, HIF3A |
| Albinism | 2 | OCA2, TYRP1 |
| Transmembrane helix | 35 | HTR2A, ADAM28, CD1D, FXYD1, FCRL6, OCA2, TRDC, ADGRD1, ADGRF4, ANO2, ACKR1, ATG9B, CNR1, CA9, CYP4B1, DCT, HS3ST2, INSRR, LAIR1, LDLRAD4, PILRA, KCNC3, KCND3, P2RX5, RXFP1, SCARA5, SHISA9, SDR42E1, SLC6A1, SYT6, TFR2, TMEM176A, TMEM176B, TREM1, TYRP1 |
| Acute phase | 2 | ORM1, SERPINA1 |
| Transmembrane | 35 | HTR2A, ADAM28, CD1D, FXYD1, FCRL6, OCA2, TRDC, ADGRD1, ADGRF4, ANO2, ACKR1, ATG9B, CNR1, CA9, CYP4B1, DCT, HS3ST2, INSRR, LAIR1, LDLRAD4, PILRA, KCNC3, KCND3, P2RX5, RXFP1, SCARA5, SHISA9, SDR42E1, SLC6A1, SYT6, TFR2, TMEM176A, TMEM176B, TREM1, TYRP1 |
| Cytoplasmic vesicle | 6 | HTR2A, HCK, AP3B2, ATG9B, PCSK2, SYT6 |
| Ion transport | 7 | FXYD1, ANO2, KCNC3, KCND3, P2RX5, SCARA5, TF |
| Ion channel | 5 | FXYD1, ANO2, KCNC3, KCND3, P2RX5 |

| Cluster 5 | | |
| --- | --- | --- |
| UP_KEYWORDS | Gene Number | Gene |
| Glycoprotein | 19 | HTR2A, BPIFB4, GPR160, KISS1R, WFDC2, ADGRD1, ANO2, CNR1, CNTNAP4, GRID1, GDF10, ITIH5, ICAM5, ISM2, HLA-G, NGF, PTPRH, ZP1, ZG16B |
| Transmembrane helix | 20 | HTR2A, GPR160, HRASLS, KISS1R, SLITRK6, ADGRD1, ANO2, CNR1, C15orf48, CLDN1, CNTNAP4, CYP4B1, GRID1, ICAM5, LRAT, HLA-G, PTPRH, SLC7A10, TMEM74B, ZP1 |
| Transmembrane | 20 | HTR2A, GPR160, HRASLS, KISS1R, SLITRK6, ADGRD1, ANO2, CNR1, C15orf48, CLDN1, CNTNAP4, CYP4B1, GRID1, ICAM5, LRAT, HLA-G, PTPRH, SLC7A10, TMEM74B, ZP1 |
| Signal | 16 | BPIFB4, SLITRK6, WFDC2, ADGRD1, CNTNAP4, GRID1, GDF10, ITIH5, ICAM5, ISM2, LRAT, HLA-G, NGF, PTPRH, ZP1, ZG16B |
| Disulfide bond | 14 | HTR2A, BPIFB4, KISS1R, WFDC2, CLDN1, CNTNAP4, GDF10, ICAM5, ISM2, HLA-G, NGF, PTPRH, SLC7A10, ZP1 |
| Protease inhibitor | 3 | WFDC2, ITIH5, NGF |
| Cell membrane | 12 | HTR2A, GPR160, HCK, KISS1R, SLITRK6, ADGRD1, ANO2, CNR1, CLDN1, CNTNAP4, GRID1, ZP1 |
| Membrane | 21 | HTR2A, GPR160, HCK, HRASLS, KISS1R, SLITRK6, ADGRD1, ANO2, CNR1, C15orf48, CLDN1, CNTNAP4, CYP4B1, GRID1, ICAM5, LRAT, HLA-G, PTPRH, SLC7A10, TMEM74B, ZP1 |
| Secreted | 8 | BPIFB4, WFDC2, GDF10, ITIH5, ISM2, NGF, ZP1, ZG16B |
| G-protein coupled receptor | 5 | HTR2A, GPR160, KISS1R, ADGRD1, CNR1 |
| Receptor | 7 | HTR2A, GPR160, KISS1R, ADGRD1, CNR1, CLDN1, GRID1 |
| Transducer | 5 | HTR2A, GPR160, KISS1R, ADGRD1, CNR1 |

| Cluster 7 | | |
| --- | --- | --- |
| UP_KEYWORDS | Gene Number | Gene |
| Signal | 9 | DCC, ST8SIA2, ACPP, CPXM1, F2RL2, COL22A1, DHRS9, EREG, SHISA2 |
| Serine protease inhibitor | 2 | SERPINB3, SERPINB4 |

| Cluster 8 | | |
| --- | --- | --- |
| UP_KEYWORDS | Gene Number | Gene |
| Microsome | 1 | CYP1A1 |
| Monooxygenase | 1 | CYP1A1 |
| Heme | 1 | CYP1A1 |
| Metal-binding | 3 | CYP1A1, MKRN3, TRIM55 |
| Iron | 1 | CYP1A1 |
| Endoplasmic reticulum | 1 | CYP1A1 |

No DEGs included in cluster 3 and cluster 6. DEGs, differentially expressed genes; UP_KEYWORDS, UniProtKB keywords
